# Effect of *Cordyceps militaris* extract containing cordycepin on the adipogenesis and lipolysis of adipocytes

**DOI:** 10.1002/2211-5463.13930

**Published:** 2024-11-21

**Authors:** Kazuya Kusama, Kodai Oka, Yumi Yashiro, Kanoko Yoshida, Hiroaki Miyaoka, Kazuhiro Tamura

**Affiliations:** ^1^ Department of Endocrine Pharmacology Tokyo University of Pharmacy and Life Sciences Japan; ^2^ Department of Biomolecular Organic Chemistry Tokyo University of Pharmacy and Life Sciences Japan

**Keywords:** adipocyte, cell senescence, cordycepin, *Cordyceps militaris*, oxidative stress

## Abstract

Obesity, a global health concern, results from an energy imbalance leading to lipid accumulation. In the present study, *Cordyceps militaris* extract (CM) and its primary component, cordycepin, were investigated to characterize their potential effects on adipogenesis and lipolysis. Treatment with CM or cordycepin reduced lipid droplets and increased hormone‐sensitive lipase activation in 3T3‐L1 cells. In a diabetic obese mouse model, CM and cordycepin lowered serum low‐density lipoprotein/very low‐density lipoprotein levels and reduced oxidative stress and cell senescence markers. Thus, cordycepin inhibits preadipocyte differentiation and promotes lipolysis, which may serve as a novel obesity treatment. Further studies, including clinical trials, are required to validate the clinical potential of cordycepin.

## Graphical abstract

*Cordyceps militaris* extract (CM) including cordycepin inhibits the differentiation into adipocytes through a decrease in peroxisome proliferator‐activated receptor (PPAR)γ and CCAAT/enhancer‐binding protein (C/EBP)β. In turn, CM also induces lipolysis via activation of hormone‐sensitive lipase (HSL). Furthermore, CM including cordycepin inhibits oxidative stress and cell senescence in adipocytes.

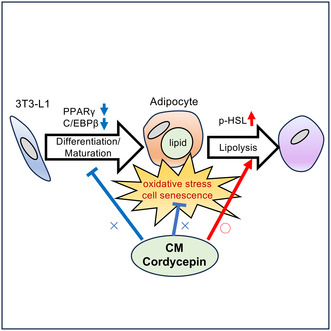

**Abbreviations**
C/EBP: CCAAT/enhancer‐binding protein
CM: *Cordyceps militaris* extract
ERK: extracellular signal‐regulated kinase
HDL: high‐density lipoprotein
HSL: hormone‐sensitive lipase
LDL: low‐density lipoprotein
mTOR: mammalian target of rapamycin
PKA: protein kinase A
PPAR: peroxisome proliferator‐activated receptor
TSNO: Tsumura Suzuki Non‐Obese
TSOD: Tsumura Suzuki Obese Diabetes
VLDL: very low‐density lipoprotein

Obesity, a prevalent condition in developed countries, escalates the risk of various diseases including type 2 diabetes, cardiovascular disease and cancer. Given its pathogenic properties, obesity has emerged as a significant target in global public health initiatives. The genesis of obesity can be traced to a sustained imbalance between energy intake and expenditure, resulting in the storage of excess energy as body lipids [1, 2]. During periods of energy surplus, white adipose tissue accumulates triglycerides, which are hydrolyzed (lipolysis) to release fatty acids for utilization by other tissues in times of energy need [3]. A decrease in lipolytic activity can lead to an accumulation of triglycerides in adipose tissue, thereby contributing to obesity and a range of metabolic disorders [3, 4]. Conversely, adipose lipase, by catalyzing the hydrolysis and cleavage of triglycerides, diglycerides and monoglycerides at various stages, play a crucial role in adipocyte lipolysis [5]. Primary lipases involved are adipose triglyceride lipase and hormone‐sensitive lipase (HSL). The activity of HSL is modulated by protein kinase A (PKA)‐catalyzed phosphorylation on serine residues. Other kinases, such as AMP‐activated protein kinase, extracellular signal‐regulated kinase (ERK)1/2, glycogen synthase kinase‐4 and Ca^2+^/calmodulin‐dependent kinase, also phosphorylate HSL to regulate its enzyme activity [6, 7, 8]. Despite extensive research on obesity and the development of anti‐obesity therapeutics, no drugs have been identified that can safely and effectively treat obesity.

*Cordyceps militaris*, a member of the *Cordyceps* genus, is a fungus that parasitizes the larvae of moths of the Lepidoptera order, subsequently forming fruit bodies [9]. The dried fruit bodies of *Cordyceps*, known for their specific anti‐fatigue activities and lack of side effects, have been utilized in traditional Asian medicine as a folk tonic agent [10, 11, 12, 13]. *Cordyceps militaris* typically contains cordycepin, ergosterol and linoleic acid as its main bioactive compounds [11]. Previous reports have indicated that the extract of *Cordyceps sinensis* induces lipolysis via hormone‐sensitive lipase activated by PKA [14], and that cordycepin inhibited the adipogenesis via inhibition of protein kinase B and activation of AMP‐activated protein kinase [15]. In our previous study, we found that the extract of *Samia Cynthia ricini*‐derived *C. militaris* (CM) inhibited androgen metabolism in an animal model of late‐onset hypogonadism and inhibited testosterone‐induced prostate hypertrophy in a benign prostate hyperplasia animal model [16]. We also identified cordycepin as the primary bioactive compounds inhibiting the proliferation of prostate cancer cells [17]. However, the effects of CM extract on adipogenesis and lipolysis have not yet been characterized. In the present study, we investigated the impact of CM on the differentiation of mouse 3T3‐L1 cells into adipocytes and its effect on lipolysis.

## Materials and methods

### Preparation of the extract from *C. militaris* fruit body (CM)

A microbial strain of *C. militaris* obtained from National Institute of Technology and Evaluation (NBRC 100741, Chiba, Japan) was cultured in SDY medium. An efficient method for the growth of fruit bodies of *C. militaris* parasitizing *Samia Cynthia ricini* (Ryoukyu‐kaso in Japanese) was established as described previously [16]. In addition, NMR experiments and analysis were performed to identify and quantify chemical components in CM, identifying cordycepin as a major component.

### Cell culture and differentiation into adipocytes

Mouse embryonic fibroblasts 3T3‐L1 (preadipocytes; JCRB Cell Bank, Osaka, Japan) were cultured in Dulbecco's Modified Eagle's Medium (DMEM/F‐12; Fujifilm Wako Pure Chemical Corp., Osaka, Japan) containing 10% fetal bovine serum, antibiotics and antimycotics, and the cells were grown at 37 °C in a humidified atmosphere containing 5% CO_2_. To induce the differentiation of preadipocytes into mature adipocytes, the 3T3‐L1 cells were cultured in the differentiation medium supplemented with 8 μg·mL^−1^ d‐biotin, 0.5 μg·mL^−1^ insulin, 400 ng·mL^−1^ dexamethasone, 44 μg·mL^−1^ 3‐isobutyl‐1‐methylxanthine, 9 ng·mL^−1^ l‐thyroxine and 3 μg·mL^−1^ ciglitazone for 3 days. Following this, the cells were cultured for an additional 3 days in basal medium, which was supplemented only with insulin. Furthermore, cells were cultured in basal medium without insulin for an additional 1 day before being subjected to analysis. The 3T3‐L1 cells were treated with CM (50 μg·mL^−1^), cordycepin (5 or 10 μg·mL^−1^; Fujifilm Wako Pure Chemical Corp.), 8‐cyclopentyl‐1,3‐dipropylxanthine (an adenosine A1 receptor antagonist; 10 μm; Sigma‐Aldrich, Tokyo, Japan) or AB928 (an adenosine A2a/b receptor antagonist; 1 μm; Selleck Chemicals, Houston, TX, USA) for 48 h.

### Oil Red O staining

3T3‐L1 cells cultured in differentiation medium were stained with Oil Red O solution to a standard protocol. Briefly, after fixation with 4% paraformaldehyde for 10 min at room temperature, the cells were incubated with 3 mg·mL^−1^ Oil red O (Sigma‐Aldrich) in 60% isopropanol for 30 min. After removing the solution, the cells were washed with 60% isopropanol and subsequently with phosphate‐buffered saline [18]. To evaluate the level of staining, cells were treated with 100% isopropanol for 10 min to dissolve the bound staining, and the absorbance was measured at 500 nm. The values, which represent the average of total intensity per culture well, were evaluated per the same number of cells in each well and shown as the relative levels to control.

### RNA extraction and quantitative reverse transcription PCR

Total RNA was extracted from cultured cells or tissues using ISOGEN reagent (Nippon Gene, Tokyo, Japan) in accordance with the manufacturer's instructions. Reverse transcription of isolated RNA was performed with the LunaScript RT SuperMix Kit (New England Biolabs, Beverly, MA, USA) and the generated cDNA was then subjected to quantitative PCR amplification using PowerUP SYBR Green Master Mix (Thermo Fisher Scientific, Waltham, MA, USA). The primers are listed in Table S1. Calibration curves were used to confirm that the amplification efficiencies of each target gene and the reference gene for glyceraldehyde 3‐phosphate dehydrogenase (*Gapdh*) were comparable. sequence detection system, version 2.3 (Thermo Fisher Scientific) was used to determine average threshold (*C*_t_) values for each target [19].

### Western blotting

3T3‐L1 cells were lysed with RIPA buffer (Thermo Fisher Scientific) in accordance with the manufacturer's instructions. The constituent proteins were separated by SDS/PAGE and transferred onto poly(vinylidene difluoride) membranes (Bio‐Rad Laboratories, Hercules, CA, USA) using a trans‐Blot Turbo (Bio‐Rad). After blocking with Bullet Blocking One (Nacalai Tesque, Kyoto, Japan), the membranes were incubated with primary antibodies specific for HSL (dilution 1 : 2000; Invitrogen, Carlsbad, CA, USA), p‐HSL (dilution 1 : 2000; Proteintech, Tokyo, Japan), ERK (dilution 1 : 2000; Cell Signaling Technology, Danvers, MA, USA), p‐ERK (dilution 1 : 2000; Cell Signaling Technology), peroxisome proliferator‐activated receptor (PPAR)γ (dilution 1 : 2000; Abcam, Cambridge, UK), C/EBPβ (dilution 1 : 2000; Cell Signaling Technology), γH2AX (dilution 1 : 2000; Cell Signaling Technology), p53 (dilution 1 : 2000; Cell Signaling Technology), p21 (dilution 1 : 2000; Cell Signaling Technology), p16 (dilution 1 : 2000; Cell Signaling Technology), or GAPDH (dilution 1 : 5000; Fujifilm Wako Pure Chemical Corp.). Immunoreactive bands were detected using an enhanced chemiluminescence kit (Merck Millipore, Burlingame, MA, USA) after incubation with horseradish peroxidase‐labeled goat anti‐rabbit or anti‐mouse IgG (dilution 1 : 5000; Vector Laboratories, Burlingame, CA, USA). Signals were detected using a C‐DiGit Blot Scanner (LI‐COR, Lincoln, NE, USA) and the band density was measured using image studio digit, version 5.2 [19].

### Measurement of glycerol in cultured media

Differentiated 3T3‐L1 adipocytes were treated with CM (50 μg·mL^−1^) or cordycepin (5 or 10 μg·mL^−1^) for 24, 48 or 72 h. The concentrations of glycerol in the medium were measured using EnzyChrom Adipolysis Assay kit (BioAssay Systems, Hayward, CA, USA).

### Animals and tissue preparation

Male TSOD (Tsumura Suzuki Obese Diabetes) mice and male TSNO (Tsumura Suzuki Non‐Obese) mice (each *n* = 5; Institute of Animal Reproduction, Ibaraki, Japan) were maintained under standard conditions with a 12 : 12‐h dark/light photocycle, at 23 ± 1 °C, with controlled humidity (55 ± 5%). TSOD mice are a multifactorial genetic model of spontaneously occurring type 2 diabetes, showing a gradual increase in blood glucose levels, hyperlipidemia, and hyperinsulinemia. TSNO mice that do not develop the symptoms such as TSOD were used as control animals. The experiments were started using 5‐week‐old mice. To explore the effect of CM or cordycepin on adipogenesis, TSOD or TSNO mice were freely fed three different diets. The control group was given a standard control diet (AIN93G‐based rodent diets; Oriental Yeast Co., Ltd, Tokyo, Japan), and two special diets were prepared by adding CM extract (0.1%) or cordycepin (0.000357%) to this control diet, and mice were fed for 3 weeks with free access to food and water. These two special diets were prepared by Oriental Yeast Co., Ltd. There was no difference in food intake among all groups. All animal‐handling protocols and surgical procedures were approved by the Institutional Animal Care Committees in Tokyo University of Pharmacy and Life Sciences (approval number: P22‐67), in compliance with institutional guidelines for the care of experimental animals, which was in accordance with internationally accepted principles (US guidelines/NIH publication). The day after the final treatment, the animals were sacrificed by cervical dislocation, and blood and adipose tissue were isolated. The collected tissues were frozen and stored in liquid nitrogen until analyses.

### Measurement of serum glucose

Serum glucose levels were measured using an enzymatic colorimetric assay (LabAssay Glucose Kit; Wako Pure Chemical Industries, Ltd, Osaka, Japan).

### Measurement of serum cholesterol

The concentrations of total, high‐density lipoprotein (HDL), low‐density lipoprotein/very low‐density lipoprotein (LDL/VLDL) cholesterol in the sera were measured using EnzyChrom HDL and LDL/VLDL Assay kit (BioAssay Systems).

### Statistical analysis

Data are expressed as the mean ± SEM and were compared using Dunnett's test in r, version 4.0.5 (R Foundation, Vienna, Austria). *P* < 0.05 was considered statistically significant.

## Results

### Effects of CM and cordycepin on the differentiation of 3T3‐L1 preadipocytes into adipocytes

**Fig. 1:**
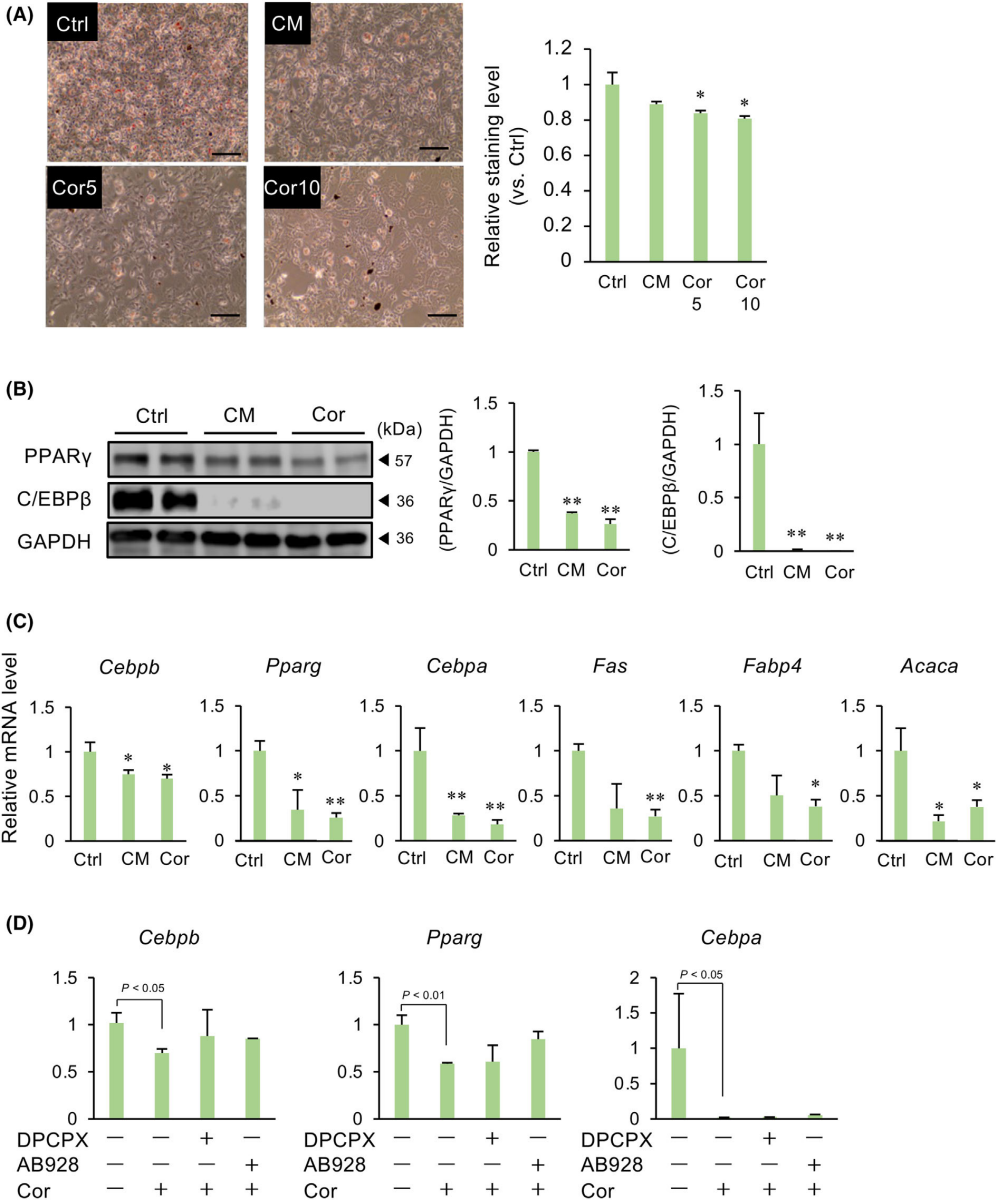
Effects of CM and cordycepin (Cor) on the differentiation of 3T3‐L1 preadipocytes into adipocytes. 3T3‐L1 cells were cultured in a differentiation medium in the presence of CM (50 μg·mL^−1^) or cordycepin (5 or 10 μg·mL^−1^) for 3 days, and then cultured in a normal medium with insulin for 3 days and in a normal medium for 1 day. (A) Lipid droplets were visualized with Oil Red O solution. The graph shows the relative level measured at 500 nm absorbance. The levels were normalized by the number of total cells each well. Values represent the mean ± SEM from three independent experiments performed in triplicates. *P < 0.05 vs. Ctrl. Scale bar = 100 μm. (B) Lysates prepared from adipocytes treated with CM or Cor were subjected to immunoblotting. Gapdh served as a loading control. The relative expression of target proteins, normalized to that of Gapdh, is shown. Values are the mean ± SEM. **P < 0.01 vs. Ctrl. (C) Changes in the expression of adipocyte differentiation markers were determined after CM or Cor (10 μg·mL^−1^) treatment using quantitative PCR. *Gapdh* was used as the reference gene. Values represent the mean ± SEM of three independent experiments performed in triplicates. *P < 0.05, **P < 0.01 vs. Ctrl. (D) Cells were cultured in a differentiation medium in the presence of 8‐cyclopentyl‐1,3‐dipropylxanthine (10 μg·mL^−1^), AB928 (1 μg·mL^−1^) and Cor (10 μg·mL^−1^) for 3 days, and then cultured normal medium with insulin for 3 days and normal medium for 1 day. Changes in the expression of adipocyte differentiation markers were determined. *Gapdh* was used as the reference gene. Values represent the mean ± SEM of three independent experiments. Dunnett's test was used to determine statistical significance.

To investigate whether CM and its main component cordycepin have effects on the differentiation of preadipocytes, 3T3‐L1 cells treated with CM (50 μg·mL^−1^) or cordycepin (5 or 10 μg·mL^−1^) in the presence of differentiation media were stained with Oil Red O. Cordycepin decreased the intensity of Oil Red O staining, and a similar trend was observed with CM (Fig. 1A). Both CM and cordycepin decreased the protein levels of differentiation markers PPARγ and CCAAT‐enhancer binding protein (C/EBP) β (Fig. 1B). Next, we examined the mRNA expression of differentiation markers: *Pparg*, *Fas*, *Fabp4* and *Acaca*, resulting in a decrease in these genes in adipocytes treated with either CM or cordycepin (Fig. 1C). Cordycepin also reduced C/EBPβ and C/EBPα expression (Fig. 1D). Cordycepin is a natural adenosine analog that binds to adenosine receptors. Therefore, we used specific antagonists of adenosine A1 and A2 receptors to determine which receptors mediate the actions of cordycepin on the expression of differentiation markers in adipocytes. None of the antagonists affected the cordycepin‐mediated inhibition of differentiation marker expression (Fig. 1D).

### Effects of CM and cordycepin on the lipolysis of adipocytes

**Fig. 2:**
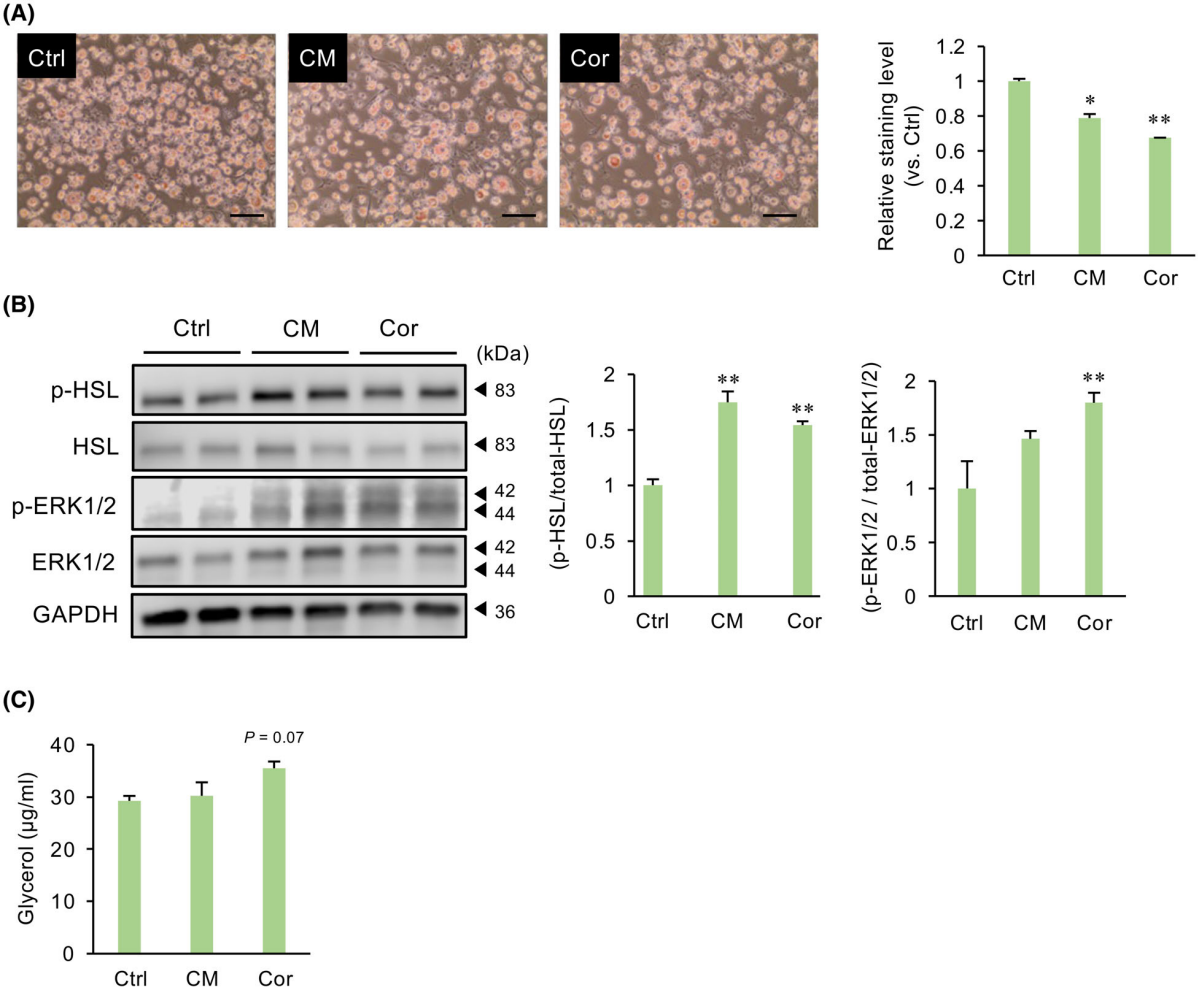
Effects of CM and cordycepin (Cor) on the lipolysis of adipocytes. Matured adipocytes were treated with CM (50 μg·mL^−1^) or Cor (10 μg·mL^−1^) for 24 h. (A) Lipid droplets were visualized with Oil Red O solution. The graph shows the relative stained level measured at 500 nm absorbance. The levels were normalized by the number of total cells each well. Values represent the mean ± SEM from three independent experiments performed in triplicates. *P < 0.05, **P < 0.01 vs. Ctrl. Scale bar = 100 μm. (B) Lysates prepared from adipocytes treated with CM or Cor were subjected to immunoblotting. GAPDH served as a loading control. The relative expression of phosphorylated HSL proteins, normalized to total HSL proteins, is shown. Values represent the mean ± SEM. **P < 0.01 vs. Ctrl. (C) Cultured media of matured adipocytes treated with CM or Cor were subjected to glycerol assay. Values represent the mean ± SEM from three independent experiments performed in triplicates. Dunnett's test was used to determine statistical significance.

We next examined whether CM and cordycepin influence lipolysis. Treatment of matured adipocytes with CM and cordycepin decreased lipid droplets (Fig. 2A). Furthermore, CM and cordycepin increased the phosphorylation of HSL and ERK1/2 (Fig. 2B). CM and cordycepin tended to increase glycerol in culture media (Fig. 2C).

### Effects of CM and cordycepin on the adipogenesis in a mouse diabetic model

**Fig. 3:**
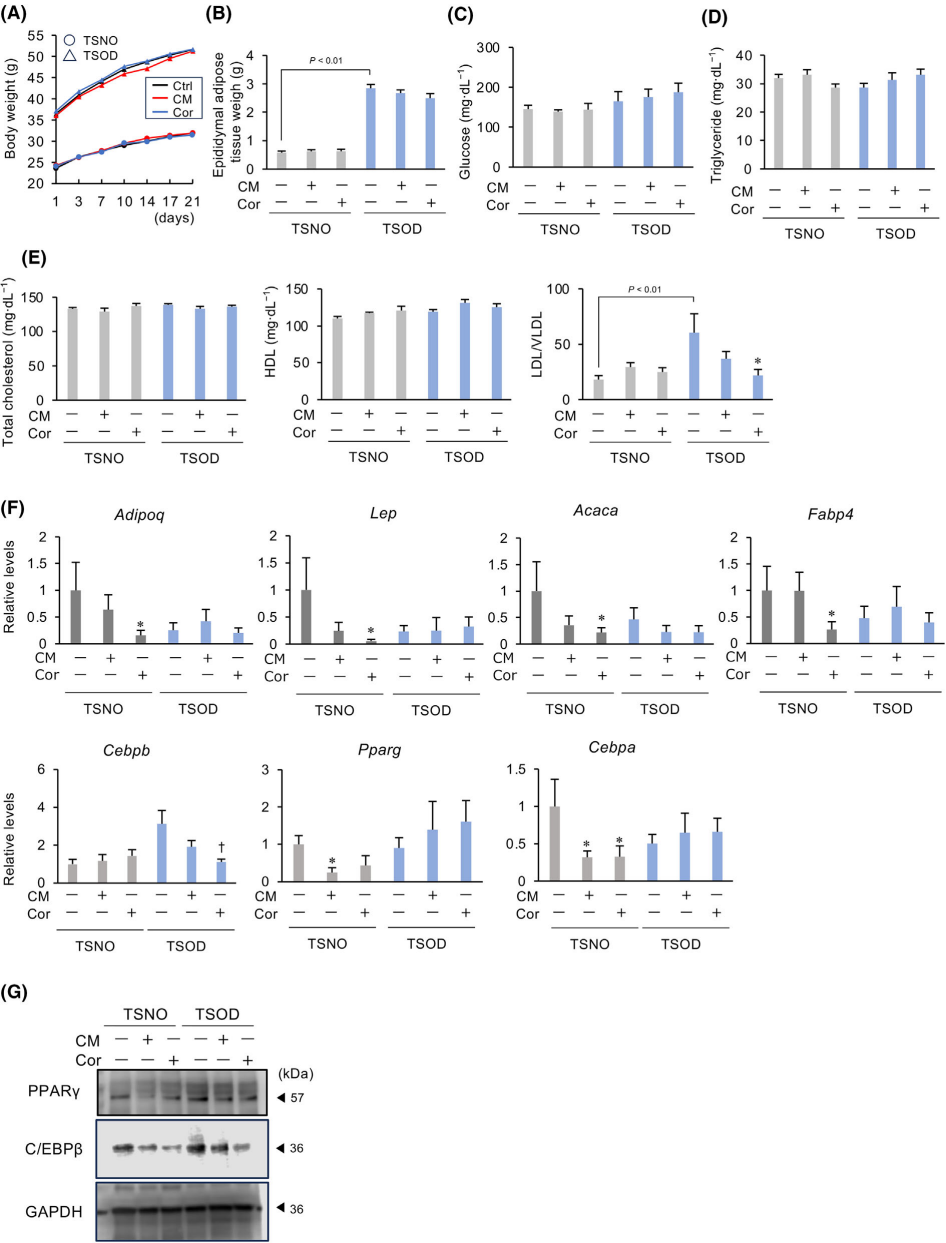
Effects of CM and cordycepin (Cor) on the adipogenesis in mouse diabetic model. TSOD or TSNO mice were fed a diet containing CM (0.1%) or Cor (0.000357%) for 21 days. The total body weight (A), the weights of adipose tissue (B), and the concentrations of serum glucose (C), triglyceride (D) or cholesterol (E) were measured on the next day after administration of CM or Cor into TSNO or TSOD mice. Data represent the mean ± SEM of five mice. (F) Changes in *Cebpb*, *Pparg*, *Cebpa*, *Adipoq*, *Lep*, *Acaca* and *Fabp4* mRNA levels in the adipose tissue. *Gapdh* was used as an internal control for RNA integrity. Data from five individual animals are shown. *P < 0.05 vs. TSNO‐Ctrl. ^†^ *P* < 0.05 vs. TSOD‐Ctrl. (G) Lysates prepared from adipose tissue were subjected to immunoblotting. GAPDH served as a loading control. Dunnett's test was used to determine statistical significance.

To further investigate the effect of CM on adipogenesis, TSOD mice, which are known to develop disease states similar to human metabolic disorders, including visceral fat accumulation, glucose intolerance, hyperlipidemia, hypertension and hyperinsulinemia, were fed a diet containing CM or cordycepin. TSNO mice were used as a control. CM and cordycepin did not alter body and fat weight in TSNO and TSOD mice (Fig. 3A,B). Furthermore, CM and cordycepin did not alter the serum levels of glucose and triglyceride (Fig. 3C,D). Although CM and cordycepin did not alter the levels of total cholesterol and HDL, they decreased LDL/VLDL levels (Fig. 3E). In the adipose tissue of TSNO mice, differentiation markers *Pparg*, *Cebpa*, *Adipoq*, *Lep*, *Acaca* and *Fabp4* were decreased by either CM or cordycepin, whereas these genes were not changed by CM and cordycepin in TSOD mice (Fig. 3F). Conversely, CM and cordycepin did not alter the expression of *Cebpb* in TSNO and also decreased such expression in TSOD mice (Fig. 3F). Similar to mRNA expression, CM and cordycepin decreased PPARγ protein in TSNO mice and decreased C/EBPβ protein in TSOD mice (Fig. 3G).

### Effects of CM and cordycepin on the oxidative stress and senescence in adipocytes

**Fig. 4:**
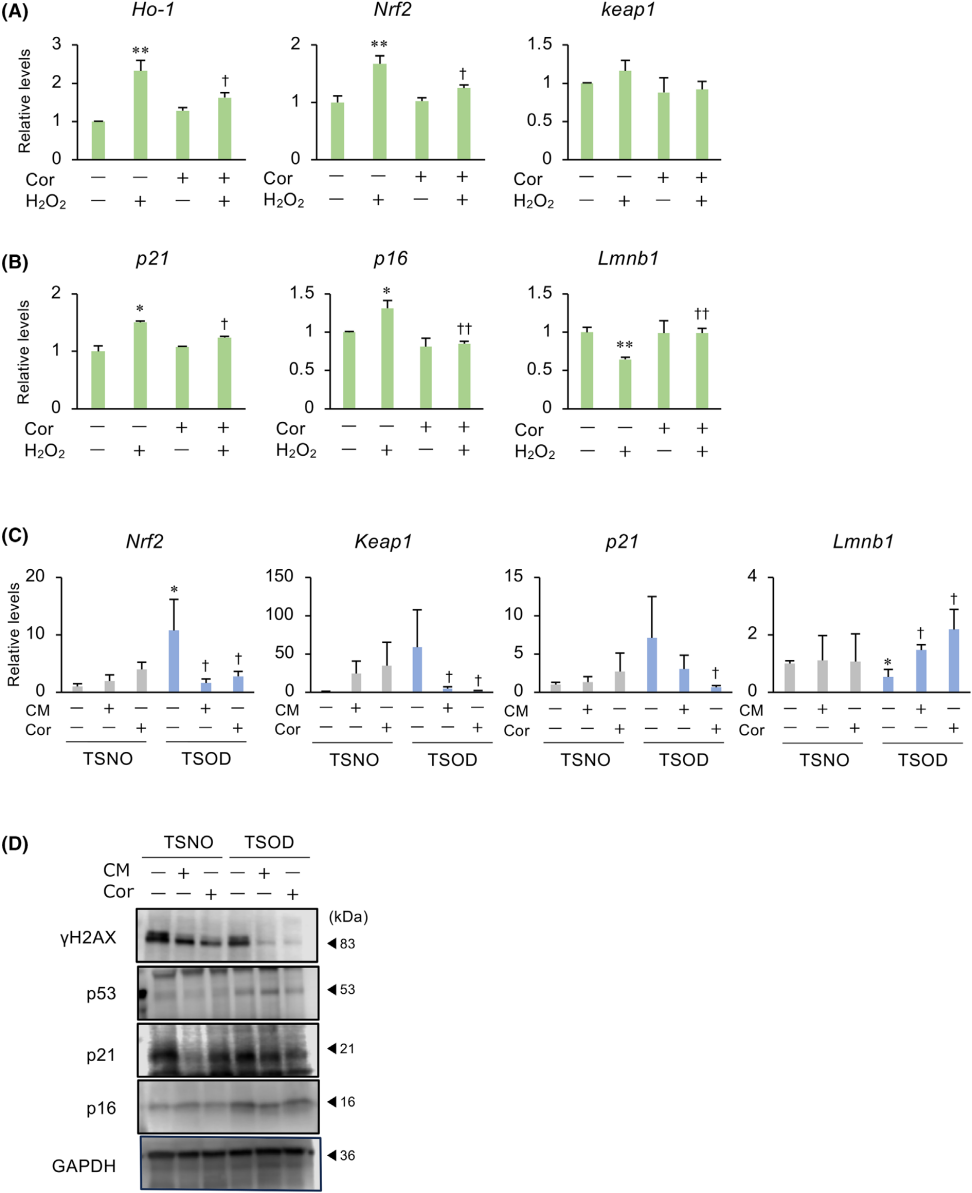
Effects of CM and cordycepin (Cor) on the oxidative stress and senescence in adipocytes. (A, B) 3T3‐L1 cells were treated with H₂O₂ (200 μm) and/or Cor (10 μg·mL^−1^) for 48 h. Changes in the expression of oxidative stress markers (A) and cell senescence markers (B) were determined using quantitative PCR. *Gapdh* was used as the reference gene. Values represent the mean ± SEM of three independent experiments performed in triplicates. *P < 0.05, **P < 0.01 vs. Ctrl. ^†^ *P* < 0.05, ^††^ *P* < 0.01 vs. H₂O₂ treatment. (C) TSOD or TSNO mice were fed a diet containing CM (0.1%) or Cor (0.000357%) for 21 days. Changes in oxidative stress marker and cell senescence marker mRNA levels in the adipose tissue. *Gapdh* was used as an internal control for RNA integrity. Data from five individual animals are shown. *P < 0.05 vs. TSNO‐Ctrl. ^†^ *P* < 0.05 vs. TSOD‐Ctrl. (D) Lysates prepared from adipose tissue were subjected to immunoblotting. GAPDH served as a loading control. Dunnett's test was used to determine statistical significance.

Levels of oxidative stress and cell senescence are high in obesity. To examine the effect of CM and cordycepin on the oxidative stress and cell senescence, 3T3‐L1 cells were treated with H_2_O_2_ to induce oxidative stress. H_2_O_2_‐upregulated expression of oxidative stress markers *Nrf2* and *Ho‐1* was decreased by cordycepin (Fig. 4A), but it did not alter *Keap1* expression. Regarding cell senescence markers expression, cordycepin decreased *p16* and *p21*, and increased *Lmnb1* expression (Fig. 4B). In the diabetic obesity mouse model, the expression of oxidative stress and senescence markers in the TSOD mouse was higher than that in the TSNO mouse, and this was reduced by CM and cordycepin (Fig. 4C). Similar to mRNA expression, γH2AX, p53, p21 and p16 expression in TSOD were higher compared to those in TSNO, which were reduced by CM and cordycepin (Fig. 4D).

## Discussion

The present study shows that CM, which includes the effective component cordycepin, inhibited the differentiation of 3T3‐L1 cells into adipocytes and promoted lipolysis in *in vitro* culture models. CM and cordycepin decreased the intensity of Oil Red O staining in 3T3‐L1 cells stimulated with a differentiation medium. Similar to Oil Red O staining, CM and cordycepin decreased the mRNA and protein levels of differentiation markers. On the other hand, CM and cordycepin reduced the content of lipid droplets in mature adipocytes. Furthermore, CM and cordycepin increased the phosphorylation of lipase HSL and glycerol in the media. These results indicate that CM, which includes the effective component cordycepin, inhibited the differentiation of preadipocytes into adipocytes and promoted lipolysis.

CM inhibited the differentiation of 3T3‐L1 cells into adipocytes. CM also decreased C/EBPβ and PPARγ. Cordycepin is reported to alleviate hepatic lipid accumulation by inducing autophagy via the PKA‐mammalian target of rapamycin (mTOR) pathway [20], and rapamycin, an inhibitor of mTOR, inhibited adipocyte differentiation and insulin action via downregulation of the expression of matured adipocyte markers: C/EBPα, PPARγ and fatty acid synthase [21]. These results suggest that CM including cordycepin inhibited the differentiation of preadipocytes via C/EBPβ‐PPARγ signaling pathway and possibly mTOR.

*In vivo* animal models showed that CM and cordycepin reduced the expression of several adipocyte markers in TSNO control mice with normal glucose metabolism. The TSOD mouse, a model of naturally occurring type 2 diabetes and obesity, is known to exhibit insulin resistance and decreased sympathetic nervous activity [22], resulting in a model with reduced adiponectin expression. Interestingly, cordycepin significantly reduced blood LDL/VLDL levels and decreased C/ebpβ expression only in TSOD mice, and CM showed a similar effect. In a previous study examining the efficacy and safety of CM in humans, liver computed tomography scans revealed that consuming 1.5 g of CM daily for 8 weeks was safe and inhibited lipid accumulation in hepatocytes, as well as the progression of fatty liver and cirrhosis [23]. These results suggest that CM and cordycepin could be beneficial in inhibiting abnormal adipocyte differentiation in type 2 diabetes and obesity.

CM and cordycepin increased the phosphorylation of HSL and ERK1/2, and increased glycerol in the cultured media. ERK1/2 regulates the phosphorylation of HSL, and phosphorylated HSL promotes the degradation of diacylglycerol into monoacylglycerol [15, 24]. Because CM and cordycepin decreased lipid droplets, CM could promote the degradation of triglyceride into diacylglycerol. Furthermore, the activity of HSL is regulated by PKA‐catalyzed phosphorylation at serine residues [15, 25]. Cordycepin is a natural adenosine analogue and binds to adenosine receptors [26]. Adenosine receptors belong to the G protein‐coupled receptor are classified four types: P1A1, P1A2a, P1A2b and P1A3. P1A2a and P1A2b bind to a Gs protein and increase the intracellular concentration of cAMP by stimulating adenylyl cyclase [27]. However, the present study showed that cordycepin did not stimulate P1A1, P1A2a and P1A2b receptors with respect to the differentiation of preadipocytes. Interestingly, recent reports indicate that the anti‐obesity effects cannot be replicated with adenosine‐related substances but are mediated by the action of adenosine transporters [14]. These findings suggest that cordycepin in CM could affect preadipocytes mediated by an adenosine transporter and activate PKA, resulting in the activation of ERK1/2‐HSL and subsequent lipolysis. Further studies of the molecular mechanisms by which cordycepin in CM inhibits adipogenesis and lipolysis using untargeted chemical biology approaches [28] might lead to the identification of cordycepin targets.

Unhealthy adipose tissue is a major source of increased reactive oxygen species production [29, 30, 31], supporting the hypothesis that increased oxidative stress in accumulated fat is an early instigator of obesity‐associated cardiometabolic complications [29, 31]. Both mature adipocytes and preadipocytes are highly sensitive to redox changes. Enlarged, dysfunctional adipocytes increase reactive oxygen species production, enhancing oxidative stress in the adipose microenvironment through oxidative damage to locally stored lipids, leading to lipoperoxidation and impairment of preadipocyte differentiation [32]. Senescence plays an important role in the development and progression of several chronic diseases, including obesity, insulin resistance and type 2 diabetes mellitus [33, 34]. The senescent phenotype can also result from stress stimuli, most commonly oxidative stress [35]. According to the Mouse Aging Cell Atlas, senescence appears to initiate earlier in fat than in other tissues [34, 36]. In obese animal models, increased DNA damage in adipocytes precedes the development of obesity, inflammation and glucose intolerance, leading to cell senescence [33]. Cordycepin has been reported to prevent radiation‐induced cell senescence via Nrf2 and Ampk in rodents [37]. We demonstrated here that CM and cordycepin inhibited the expression of oxidative stress markers, including Nrf2, and senescence markers in adipocytes. These findings suggest that CM including cordycepin may ameliorate obesity and type 2 diabetes mellitus by inhibiting oxidative stress and cell senescence in adipose tissues.

In conclusion, we identified cordycepin as the main effective component in the extract of *C. militaris* parasitizing *Samia Cynthia ricini* (CM), which inhibited the differentiation of preadipocytes into adipocytes and promoted lipolysis by partially inhibiting oxidative stress and cell senescence. However, further pre‐clinical and clinical studies are required to confirm the results obtained in the present study. Clarification of the molecular mechanisms by which cordycepin in CM inhibited adipogenesis and lipolysis might lead to the identification of novel treatments for obesity.

## Conflicts of interest

The authors declare that they have no conflicts of interest.

### Peer review

The peer review history for this article is available at https://www.webofscience.com/api/gateway/wos/peer‐review/10.1002/2211‐5463.13930.

## Author contributions

KK and KT conceived and designed the experiments. KK, KO, YY, KY and KT performed experiments and analyzed data. KK and KT wrote the manuscript. KK, KO, YY, KY, HM and KT coordinated the project and contributed to the data analysis and interpretation of the results. All authors approved the final version of the manuscript submitted for publication.

## Supporting information


**Table S1.** Primers for real‐time PCR.

## Data Availability

The data that support the findings of this study are available from the corresponding author upon reasonable request.
