# Supplementary material for: Effect of Cordyceps militaris extract containing cordycepin on the adipogenesis and lipolysis of adipocytes
Source: FEBS Open Bio. 2024 Nov 21;15(2):335–45. doi: 10.1002/2211-5463.13930 (PMC11788751; doi:10.1002/2211-5463.13930)
Supplement: Supplementary file 1 — Table S1. Primers for real‐time PCR. [file FEB4-15-335-s001.docx]

**Table S1. Primers for real-time PCR.**

| **Name**  **Accession No.** |  | **Sequence** |  | **Product length (bp)** |
| --- | --- | --- | --- | --- |
| Gapdh | F: 5'- | CATCACTGCCACCCAGAAGACTG | -3' | 153 |
| NM_001289726.1 | R: 5'- | ATGCCAGTGAGCTTCCCGTTCAG | -3' |  |
| Pparg | F: 5'- | GTACTGTCGGTTTCAGAAGTGCC | -3' | 102 |
| NM_001127330.2 | R: 5'- | ATCTCCGCCAACAGCTTCTCCT | -3' |  |
| Cebpa | F: 5'- | GCAAAGCCAAGAAGTCGGTGGA | -3' | 126 |
| NM_007678.3 | R: 5'- | CCTTCTGTTGCGTCTCCACGTT | -3' |  |
| Fas | F: 5'- | CTGCGATTCTCCTGGCTGTGAA | -3' | 130 |
| NM_007987.2 | R: 5'- | CAACAACCATAGGCGATTTCTGG | -3' |  |
| Fabp4 | F: 5'- | TGAAATCACCGCAGACGACAGG | -3' | 125 |
| NM_024406.4 | R: 5'- | GCTTGTCACCATCTCGTTTTCTC | -3' |  |
| Acaca | F: 5'- | GTTCTGTTGGACAACGCCTTCAC | -3' | 120 |
| NM_133360.3 | R: 5'- | GGAGTCACAGAAGCAGCCCATT | -3' |  |
| Adipoq | F: 5'- | AGATGGCACTCCTGGAGAGAAG | -3' | 156 |
| NM_009605.5 | R: 5'- | ACATAAGCGGCTTCTCCAGGCT | -3' |  |
| Lep | F: 5'- | GCAGTGCCTATCCAGAAAGTCC | -3' | 131 |
| NM_008493.3 | R: 5'- | GGAATGAAGTCCAAGCCAGTGAC | -3' |  |
| Cebpb | F: 5'- | CAACCTGGAGACGCAGCACAAG | -3' | 113 |
| NM_001287738.1 | R: 5'- | GCTTGAACAAGTTCCGCAGGGT | -3' |  |
| Nrf2 | F: 5'- | CAGCATAGAGCAGGACATGGAG | -3' | 107 |
| NM_010902.4 | R: 5'- | GAACAGCGGTAGTATCAGCCAG | -3' |  |
| Keap1 | F: 5'- | ATCCAGAGAGGAATGAGTGGCG | -3' | 123 |
| NM_016679.4 | R: 5'- | TCAACTGGTCCTGCCCATCGTA | -3' |  |
| Ho-1 | F: 5'- | CACTCTGGAGATGACACCTGAG | -3' | 115 |
| NM_010442.2 | R: 5'- | GTGTTCCTCTGTCAGCATCACC | -3' |  |
| p16 | F: 5'- | TGTTGAGGCTAGAGAGGATCTTG | -3' | 114 |
| NM_009877.2 | R: 5'- | CGAATCTGCACCGTAGTTGAGC | -3' |  |
| P21 | F: 5'- | TCGCTGTCTTGCACTCTGGTGT | -3' | 124 |
| NM_007669.5 | R: 5'- | CCAATCTGCGCTTGGAGTGATAG | -3' |  |
| Lmnb1 | F: 5'- | AGGAAGAGCTGGAGCAGACCTA | -3' | 150 |
| NM_010721.2 | R: 5'- | GCAGGTTAGAGAGCTGTGAGGA | -3' |  |

F: Forward, R: Reverse.
